# Consequences of Exposure to War Violence: Discriminating Those with Heightened Risk for Aggression from Those with Heightened Risk for Post-Traumatic Stress Symptoms

**DOI:** 10.3390/ijerph20126067

**Published:** 2023-06-06

**Authors:** L. Rowell Huesmann, Eric F. Dubow, Paul Boxer, Cathy Smith, Khalil Shikaki, Simha F. Landau, Shira Dvir Gvirsman

**Affiliations:** 1 Research Center for Group Dynamics, Institute for Social Research, The University of Michigan, 426 Thompson St., Ann Arbor, MI 48106-1248, USA; huesmann@umich.edu (L.R.H.); pboxer@rutgers.edu (P.B.); smithcat@umich.edu (C.S.); 2 Department of Psychology, Bowling Green State University, Bowling Green, OH 43403, USA; 3 School of Arts and Sciences, Psychology Department, Rutgers University, 101 Warren Street, Newark, NJ 07102, USA; 4 Palestinian Center for Survey and Policy Research, Off Irsal Street, Ramallah P.O. Box 76, Palestine; kshikaki@pcpsr.org; 5 Faculty of Law, Institute of Criminology, The Hebrew University of Jerusalem, Mt. Scopus, Jerusalem 9190501, Israel; simha.landau@mail.huji.ac.il; 6 Department of Communication Studies, Tel-Aviv University, Tel Aviv 6997801, Israel; shirad@tauex.tau.ac.il

**Keywords:** ethnic–political violence, youth, emotional desensitization, post-traumatic stress symptoms

## Abstract

Chronic exposure to ethnic–political and war violence has deleterious effects throughout childhood. Some youths exposed to war violence are more likely to act aggressively afterwards, and some are more likely to experience post-traumatic stress symptoms (PTS symptoms). However, the concordance of these two outcomes is not strong, and it is unclear what discriminates between those who are at more risk for one or the other. Drawing on prior research on desensitization and arousal and on recent social–cognitive theorizing about how high anxious arousal to violence can inhibit aggression, we hypothesized that those who characteristically experience *higher* anxious arousal when exposed to violence should display a lower increase in aggression after exposure to war violence but the same or a higher increase in PTS symptoms compared to those low in anxious arousal. To test this hypothesis, we analyzed data from our 4-wave longitudinal interview study of 1051 Israeli and Palestinian youths (ages at Wave 1 ranged from 8 to 14, and at Wave 4 from 15–22). We used the 4 waves of data on aggression, PTS symptoms, and exposure to war violence, along with additional data collected during Wave 4 on the anxious arousal participants experienced while watching a very violent film unrelated to war violence (*N* = 337). Longitudinal analyses revealed that exposure to war violence significantly increased both the risk of subsequent aggression and PTS symptoms. However, anxious arousal in response to seeing the unrelated violent film (measured from skin conductance and self-reports of anxiety) moderated the relation between exposure to war violence and subsequent psychological and behavioral outcomes. Those who experienced greater anxious arousal while watching the violent film showed a weaker positive relation between amount of exposure to war violence and aggression toward their peers but a stronger positive relation between amount of exposure to war violence and PTS symptoms.

## 1. Introduction

Our research on Israeli and Palestinian youth has shown that exposure to ethnic–political conflict and violence has deleterious impacts on them [1,2,3], and we have drawn on our theoretical model of psychological mechanisms that account for exposure to violence in general to inform our research on exposure to ethnic-political violence [4,5,6]. Other research teams have similarly shown the negative impacts on youth of exposure to ethnic-political violence around the world [7,8,9,10,11,12,13]. The impacts are wide ranging, but as Qouta et al. [11] noted, researchers most often have been concerned with effects on internalizing symptoms and dysfunctional behaviors related to these symptoms. Exposure to extreme ethnic–political violence seems to lead to intrusion symptoms associated with the persistent exposure (e.g., recurrent nightmares about the exposure); avoidance of stimuli associated with the exposure (e.g., avoidance of external reminders); alterations in cognition and mood (e.g., negative emotional states, inability to experience positive emotions); and alterations in arousal and reactivity associated with the exposure (e.g., hypervigilance, destructive behavior, sleep disturbance) among children (e.g., for studies in the Middle East, [11,12,13,14,15,16,17]). These are recognized as hallmark criteria of post-traumatic stress disorder [18]. Indeed, across studies in the Middle East, observed rates of diagnosed PTSD are generally between 20% and 50%, depending on the degree of exposure to violence, ethnic group (lower among Israeli children, higher among Palestinian children), and time since the end of exposure (rates decline over time). Consequently, exposure to ethnic–political violence can be considered a traumatic event for many people that consequently leads to typical post-traumatic stress symptoms in the exposed person.

However, individual differences are substantial. For example, ethnic–political violence exposure does not predict post-traumatic stress symptoms for a significant proportion of individuals. That does not mean that there are no deleterious consequences. Numerous studies have now shown that for many people, exposure to ethnic–political violence is followed by an increased risk of interpersonal aggressive or violent behavior [2,3,18,19,20,21,22]. This is not surprising given the large research literature showing that repeated exposures to interpersonal violence in the family, in peers, or even in the media increases the risk of aggressive and violent behavior in the exposed person. The evidence is particularly compelling that individuals who are exposed to more violence around them during childhood, whether in their actual environment [5,23,24,25] or in mass media [26,27,28], subsequently behave more aggressively.

A number of processes have been hypothesized to explain these effects. Observational learning [29] as elaborated over the years [30,31] has been the primary process hypothesized to contribute to both long-term and short-term effects, although excitation transfer [32] and priming [33,34] have also been shown to contribute to short-term effects. Similarly, a number of theorists have proposed that so-called “desensitization” could contribute to long-term effects [27]. Desensitization is the process through which an observer of violence becomes habituated to it and no longer experiences a negative emotional reaction to it.

According to the theory of emotional desensitization, with repeated exposure to violence people become “numb” to the violence, i.e., they experience less anxiety with each new exposure. This “numbness”, in turn, is thought to make people more accepting of violence and even more violent. Why would it make them even more violent? According to Huesmann’s social–cognitive evaluation theory [28,31], it is because people exposed to persistent violence then do not “feel bad” about the outcomes of aggressive scripts that they consider (consciously or unconsciously) using. In contrast, experiencing anxiety repeatedly in response to seeing violence generalizes to experiencing anxiety when considering using an aggressive script. That anxiety at the thought of behaving aggressively then inhibits both the encoding and use of aggressive scripts to solve social problems. It follows that individuals who characteristically show less negative emotional reaction when exposed to violence would be more prone to acquire aggressive scripts when exposed to violence and to use them subsequently. On the other hand, individuals who characteristically show more negative emotional reactions when exposed to violence would be less prone to acquire aggressive scripts when exposed to violence and to follow those scripts afterwards, but they would be more prone to experience post-traumatic stress symptoms afterwards.

Preliminary support for this thinking has been provided by a few laboratory studies that examined the relations between emotional reactions to observed violence in film clips and subsequent aggressive behavior. Titus [35] showed that anxious arousal to violent film clips was significantly negatively correlated with subsequent aggression in a competitive reaction time task among American college students. Similarly, Kirwil [36] and Krahe et al. [37] both showed that lower anxious arousal to violence in a film clip was predictive of higher aggression in a subsequent laboratory aggression task among college students. However, there seem to be no studies that have examined how the relation between exposure to ethnic–political violence and subsequent aggression or subsequent PTS symptoms is moderated by a person’s characteristic emotional reactions to exposure to violence.

### The Current Study

These data were collected during our Israeli–Palestinian Exposure to Violence Study [1,3,19,38,39] and provide an opportunity to test the hypothesis that characteristic emotional reactions to observing violence can discriminate between those prone to displaying more PTS symptoms after exposure to ethnic–political violence and those prone to displaying more aggressive behavior. In prior publications, we have shown that cumulative early exposure to ethnic–political conflict and violence predicts concurrent and subsequent aggression and post-traumatic symptoms [1,2,14,15,19]; that the effect of early violence exposure on later aggression is mediated by social–cognitive variables and emotional distress [3]; and that non-punitive parenting and child self-esteem moderate the otherwise negative effects of violence exposure on post-traumatic stress symptoms [39]. This is the first publication in which we examine the potential moderating role of emotional arousal to violence in the relation between exposure to ethnic–political violence and subsequent outcomes.

This longitudinal study consisted of 4 waves of observations between 2007 and 2015 on 451 Israeli Jewish youths and 600 Palestinian youths. The youths were from 3 initial age cohorts in 2007 (modal ages 8, 11, and 14). We interviewed youths and their parents each year for 3 consecutive years (2007, 2008, and 2009–2010), and we interviewed them again for a fourth wave between 2014 and 2015, when the youths were approximately 14, 17, and 20 years of age, respectively; a randomly selected subsample of 562 youths was again assessed in a Wave 4 of interviews.

## 2. Methods

### 2.1. Sample

Palestinian and Israeli Jewish youths (*N* = 1051 at Wave 1; three age cohorts: ages 8, 11, and 14) and their parents completed three annual interviews between 2007 and 2010. A random subsample of 400 Palestinian youths and their parents and 162 Israeli Jewish youths and their parents later completed a 4th wave of interviews in 2014–2015; a subsample of 337 of these received an assessment of their characteristic level of anxious-arousal to observed violence at that time, as described below.

**Palestinian sample.** In Wave 1, the Palestinian sample included 600 children: 200 8-year-olds (101 girls, 99 boys), 200 11-year-olds (100 girls, 100 boys), and 200 14-year-olds (100 girls, 100 boys); we also interviewed a parent of each youth (98% were mothers). Residential areas were sampled to be representative of the general population of the West Bank (64%) and Gaza (36%) [14,19]; 10% of families who were initially approached declined to participate. Staff from the Palestinian Center for Policy and Survey Research conducted the sampling and interviews.

Over 99% (599/600) of the parents were Muslim, and 99% were married. One-third of the parents reported having achieved at least a high school degree. On average, there were 4.89 (*SD* = 1.86) children in the home. These statistics are representative of the general population of Palestinians [40].

At Wave 2, 590 children and their parents were re-interviewed (98% resampling rate), and at Wave 3, 572 were re-interviewed (95% resampling rate). Student’s *t*-tests showed that by Wave 3, parents of non-resampled children rated their children lower in aggression at Wave 1, but attrition was not related to Wave 1 child age, child gender, parents’ education, exposure to political conflict/violence, or child self-reported aggression and emotional distress.

In Wave 4, because of funding constraints, we re-interviewed a random sample of participants (400 Palestinian youths: 199 females, 201 males; 132 14-year-olds, 140 17-year-olds, and 128 20-year-olds). Participants who were interviewed and not interviewed in Wave 4 showed no differences in parental education at Wave 1, child gender, age, Wave 1 child aggression, or exposure to ethnic–political violence. A subsample of 258 of these 400 received an assessment of their characteristic level of anxious arousal to observed violence at that time, as described below. The subsample excluded youths who had been originally in the 8-year-old cohort because of concerns about negative impacts of the assessment on young youths.

**Israeli sample.** Demographic characteristics of the Israeli Jewish sample were as follows: 451 children (151 8-year-olds, of whom 79 were girls and 72 were boys); 150 11-year-olds, of whom 73 were girls and 77 were boys; and 150 14-year-olds, of whom 94 were girls and 56 were boys. We also interviewed one of their parents (87% were mothers).

We oversampled high-risk regions because the level of ethnic–political violence is relatively low in the major population centers of Israel. Families were sampled using the following methods: random phone calls and door-to-door cluster sampling based on neighborhoods, in addition to non-probability sampling (participants’ recommendations for families in the age range for the study) [15]. Fifty-five percent of families who were approached agreed to participate. Staff from the Machshov Survey Research Institute conducted the sampling and interviews.

The demographic characteristics of the sample were as follows: 91% of the parents were married; over 80% had graduated from high school; and, on average, there were 3.59 (*SD* = 1.83) children in the home.

In Wave 2, the re-sampling rate was 68% (*n* = 305), and in Wave 3 it was 63% (*n* = 282). Attrition was mostly due to “refusals”; parents viewed the monetary reimbursement as insufficient (there were substantial changes in exchange rates from Wave 1, so the monetary incentive was lower). Attrition analyses showed that in comparison to Wave 1, at Wave 3 children exhibited lower levels of severe physical aggression and emotional distress; parents at Wave 3 had lower levels of education compared to Wave 1. However, attrition was not related to child age, gender, or ethnic–political political violence exposure.

In Wave 4, similar to the Palestinian sample, a random sample of Israeli Jewish participants were re-interviewed (*N* = 162, 90 of whom were females and 72 of whom were males; 56 14-year-olds, 62 17-year-olds, and 44 20-year-olds). Most parents (80–85%) had achieved at least a high school degree. Regarding attrition, the youths interviewed and not interviewed at Wave 4 were similar in gender, age, and Wave 1 aggression, but youths who were re-interviewed at Wave 4 had been exposed at Wave 1 to slightly more ethnic–political violence (*t*(898) = 1.77, *p* < 0.08), and parents at Wave 1 had higher levels of educational attainment (*t*(897) = 9.21, *p* < 0.001). A subsample of 79 of these 162 also received an assessment at that time of their characteristic level of anxious arousal to observed violence at that time, as described below. The subsample excluded youths who had been originally in the 8-year-old cohort because of concerns about negative impacts of the assessment on young youths.

### 2.2. Consent and Interview Procedures

The study was approved by the institutional review boards at the University of Michigan, the Hebrew University of Jerusalem, and the Palestinian Center for Policy and Survey Research. One parent and one child in each family were interviewed. Written parental consent and child assent were obtained. Each family was compensated at the region’s equivalent rate of USD 25 for the 1 h interview during each of the first three waves. At Wave 4, Israeli families were compensated at USD 65 per child and USD 40 per parent; Palestinian families were compensated at USD 30 per child and USD 30 per parent. Because Wave 4 occurred four years after Wave 3, we increased the monetary incentives and calibrated them to average income differences between the two regions.

We designed many procedures to ensure accurate and honest responding. In our consent forms, we indicated that code numbers, not names, would be included on surveys; only the survey company would house the list linking the names and code numbers for the purposes of contacting participants for future interviews and to link their responses at each wave. We stressed that if any questions caused discomfort, participants could skip those questions, and they could discontinue their participation at any point. We conducted interviews in the participants’ homes using a well-respected survey company from each region. Interviewers were from the same ethnic group as each participant.

### 2.3. Measures

We presented the measures in the native languages in the two regions. We translated and back-translated the English measures using native-speaking research staff. We presented the measures in the same order across waves of data collection, in the order of our presentation of the measures below.

**Demographic information:** Parents provided information on their child’s age and sex, as well as an index of each parent’s level of education (which we coded as follows: 1 = illiterate to 10 = doctorate or law degree); we computed the average of the two parents’ educational levels.

**Exposure to ethnic–political conflict and violence in W1–W3:** In Waves 1–3, parents of the 8-year-old cohort reported on their children’s ethnic–political violence exposure, whereas youths in the 11- and 14-year-old cohorts provided their own reports. At Wave 4, youths in all cohorts provided self-reports. The measure includes 24 items adapted from Slone, Lobel, and Gilat [41]. The events represent the following domains of political conflict and violence: loss of, or injury to, a friend or family member (5 events), e.g., “Has a friend or acquaintance of yours been injured as a result of political or military violence?”; non-violent events (6 events), e.g., “How often have you spent a prolonged period of time in a security shelter or under curfew?”; self or significant others participating in political demonstrations (3 events), e.g., “How often have you known someone who was involved in a violent political demonstration?”; witnessing actual violence (4 events), e.g., “How often have you seen right in front of you [members of your ethnic group] being held hostage, tortured, or abused by [members of the other ethnic group]?”; and witnessing media portrayals of violence (6 events), e.g., “How often have you seen video clips or photographs of injured or martyred [members of your ethnic group] on stretchers or the ground because of an attack by [members of the other ethnic group]?” Respondents indicated the extent to which the child experienced the specific event in the past year along a 4-point scale (0 = never to 3 = many times). The score on the index is the average of the responses to the 24 events. The events often occur independently of each other, so we prefer to call the score an index of exposure rather than a scale. Nevertheless, the events intercorrelate enough that the internal consistency reliability of the index ranged from 0.70 to 0.85 across ethnic groups, age cohorts, and genders.

Wave-to-wave correlations of exposure ranged from *r* = 0.47 to *r* = 0.59 for Palestinians and from *r* = 0.44 to *r* = 0.56 for Israeli Jews. Our theory is not concerned with the effect of a one-time exposure or short-term fluctuations in exposure to war or ethnic–political violence, but with the effects of repeated, cumulative exposures. Consequently, we computed a mean score of exposure across waves reflecting cumulative exposure during Waves 1–3 to use as a predictor of Wave 4 outcomes.

**Severe physically aggressive behavior in W4:** All participants in Wave 4 were administered a 3-item *index* of their frequency of committing Severe Physical Aggression in the last year [3,42]. Respondents indicated how often in the last year they had engaged in each of three physically serious aggressive behaviors for adults along a 4-point scale (0 = never to 3 = 5 or more times). The total index score is the mean of these 3 items: “In the past year how often have you choked someone?”; “In the past year how often have you threatened or actually cut another person with a knife?”; and “In the past year, how often have you threatened or actually shot at another person with a gun?” We have shown that this is a valid measure of severe physical aggression across age, gender, and ethnic group [3]. Based on other, similar measures of seriously violent behavior (e.g., parent-to-child aggression [43]), and due to high skewness on some items, we view this score as an index of the frequency of severe physical aggression engaged in during the past year rather than a scale, as the three behaviors, while all seriously physically violent, may occur independently of each other and need not be correlated.

**Early Aggressiveness in W1:** We assessed each youth’s aggression in Wave 1 using three well-validated measures and then computed a factor-weighted, structurally invariant overall aggression score for each participant. First, youths were administered a self-report measure of their physical aggressiveness used in previous studies [42,44]. Respondents indicated how often in the last year they had engaged in four physically aggressive behaviors along a 4-point scale (0 = never to 3 = 5 or more times), e.g., “How often have you punched or beaten someone?” and “How often have you choked someone?” Second, youths were given a modified, 10-item self-report version of the Peer Nomination of Aggression Inventory [44], based on the original peer-rated index [45,46]. Children provided ratings on a 4-point scale ranging from 0 (never) to 3 (almost always) on items measuring verbal aggression (e.g., “How often do you say mean things?”), physical aggression (e.g., “How often do you push or shove other people/kids?”), indirect aggression (e.g., “How often do you make up stories and lies to get others into trouble?”), and acquisitive aggression (e.g., “How often do you take others’ things without asking”). The scale score is the mean of the 10 item scores, and the reliability was α = 0.80. Third, parents reported on their children’ behavior via the 20-item aggression subscale of the Child Behavior Checklist (CBCL) [47]. Parents rated the extent to which children displayed each aggressive behavior during the 6 months prior to assessment (e.g., “argues a lot”, “threatens people”, and “gets in many fights”) on a 3-point scale (0 = “not true”, 1 = “somewhat or sometimes true”, and 2 = “very true or often true”). The scale score is the mean of the 20 item scores, and the reliability is high (α = 0.91).

We then combined these three well-validated measures of aggression into one overall composite measure, following a procedure we have used before [48]. We computed a structural equation measurement model combining the three measures. The model fit the data well (χ^2^ = 4.1 to 6.4, *p* > 0.70, *df* = 9, CFI = 1, *RMSEA* = 0) and was adequately invariant across gender, age, and ethnic group. The factor score coefficients that the model yielded were 0.176 for the parents’ reports on the Child Behavior Checklist (aggression subscale), 0.176 for self-reports on the general aggression scale, and 0.473 for self-reports on the physical aggression scale. Consequently, scores on the factor-weighted composite aggression scale could range from 0 if a participant displayed no aggression on any scale to 2.3.

**Post-Traumatic Stress (PTS) Symptom Measure in W4:** We used Weathers et al.’s [49] scale (civilian version) of the frequency of 17 post-traumatic stress symptoms as our assessment of a participant’s level of PTS symptoms at Wave 4. The participant was asked, “How much in the past month have you…” been “bothered by” or “experienced” each symptom. The 17 symptoms are taken from each of the four *DSM-IV* criteria for post-traumatic stress, i.e., “Re-experiencing the event (e.g., intrusive memories or dreams)”, “Avoidance of stimuli associated with the traumatic event,“ “Emotional numbing”, and “Increased arousal (e.g., hypervigilance, irritability, sleep problems)”. The participant’s possible responses for each of the 17 questions are: 1 = “Not at all”, 2 = “A little bit”, 3 = “Moderately”, 4 = “Quite a bit”, and 5 = “Extremely”. We used the mean of the 17 items as the scale score. Thus, the scale score for PTS symptoms could range from 0 to 5. The internal consistency reliability of the scale in this study was very high, at α = 0.93.

**Early Post-traumatic Stress Symptoms in W1:** Children completed 9 items (α = 0.75) from the Child Post-Traumatic Stress Symptoms Index [50]. The items follow the major PTSD symptom clusters in the *DSM-IV*. The scale was administered immediately after the exposure to conflict and violence items. Youths were asked to think about these events and then asked, “Tell me how often you had these feelings and thoughts in the past month… 0 = never, 1 = hardly ever, 2 = sometimes, 3 = a lot”. We chose three items from each of three symptom cluster subscales: re-experiencing the event (e.g., “You have upsetting thoughts, pictures, or sounds of what happened come into your mind when you do not want them to.”); avoidance of stimuli associated with the event (e.g., “You try not to talk about, think about, or have feelings about what happened.”); and increased arousal (e.g., “When something reminds you of what happened, you have strong feelings in your body like heart beating fast, headaches, or stomach aches.”). Scores are the sum of responses to the items.

**Anxious Arousal to Violence:** As described in the introduction, “Anxious Arousal to Violence” is a concept that has evolved out of years of psychological research on media effects and in particular on desensitization to violence. Anxious arousal involves both a physiological arousal in response to a violent stimulus and a cognitive interpretation of that arousal as representing fear or anxiety at some level. Thus, it must be assessed as a product of a measure of a person’s physiological arousal to a violent stimulus and the person’s interpretation of that arousal. In this study we assessed each participant’s physiological arousal by measuring their skin conductance, which is known to increase with arousal. We measured their cognitive interpretation of that physiological arousal by asking them about it. Specifically, we assessed their anxious arousal to violence with a procedure used in several prior studies by these investigators and others [35,37,51,52]. The procedure involves exposing the participant to a short video clip with some highly violent scenes while assessing their skin conductance response (SCR). As with previous studies, we exposed each participant to a 4 min scene from the movie *Casino* that showed a man being beaten to a bloody pulp in a wheat field. We assessed the participant’s skin conductance response for a minute before the scene started (called the baseline period) and then during the entire scene. We then computed the participant’s mean skin conductance response during the baseline period and subtracted that from the participant’s mean skin conductance response during the two 30 s periods when the greatest amounts of blood, gore, and violence were shown. This difference in SCR, which ranged from 0 to 6, is the measure of the amount of physiological arousal the violent scene caused. However, the physiological arousal might be experienced by the participant as several different emotions. To determine which emotions a participant was feeling, the participant was asked, immediately after the clip ended, to estimate how much “anxiety, fear, horror, pleasure, and amusement” he or she felt during the film clip, also rated on a 0 to 6 scale from “0 = none to 6 = very much”. Because the physiological arousal of some participants might represent pleasure or amusement at seeing the violence, we asked for those other ratings. However, our theoretical interest was in how much anxious arousal each participant experienced by being exposed to such violence. Because the range of correlations among participants’ ratings of anxiety, fear, and horror was *r* = 0.65 to *r* = 0.77 and because they all represented negative emotions related to anxiety, we computed a mean of these 3 ratings for each participant and used that score as the best measure of the extent to which the participant interpreted what he or she was feeling during the violent scenes as anxiety. Then, we computed a total anxious arousal to violence score for each participant by multiplying their mean increase in physiological arousal during the violent scene (scaled 0 to 6) by the extent to which they interpreted what they felt while they watched as anxiety (also scaled 0 to 6). This score, which could range from 0 to 36, was defined as the person’s “characteristic anxious arousal to violence”. It is interpreted as an individual difference trait variable, relatively constant across states. We obtained these measures for 258 of our resampled Palestinian youths and for 79 of our resampled Israeli Jewish youths, giving us a total sample of 337 for whom we had measures of their “characteristic anxious arousal to violence”. As noted, youths who had been in the 8-year-old cohort at Wave 1 were excluded because of concerns about the potential negative impact of the video upon them. The anxious arousal scores ranged from 0–35.18, with a median of 1.98, a mean of 4.36, and a skewness of 1.96.

## 3. Results

The means and standard deviations and skewness values for the four key variables measured in the study for the overall sample of *N* = 1051 are shown in Table 1. Because the measure of Severe Physical Aggression in Wave 4 (frequency of choking, stabbing, or shooting) is highly skewed (4.05), the log of this measure is used for all analyses treating it as a continuous variable.

The correlations among the four key study variables are shown in Table 2. One can see that both the Post-Traumatic Stress Symptom score and the Frequency of Severe Physical Aggression score (choking, stabbing, or shooting) in Wave 4 are significantly positively correlated with the average yearly frequency of Exposure to Ethnic–Political Violence experienced 4 to 8 years earlier in Waves 1–3.

**Figure 1:**
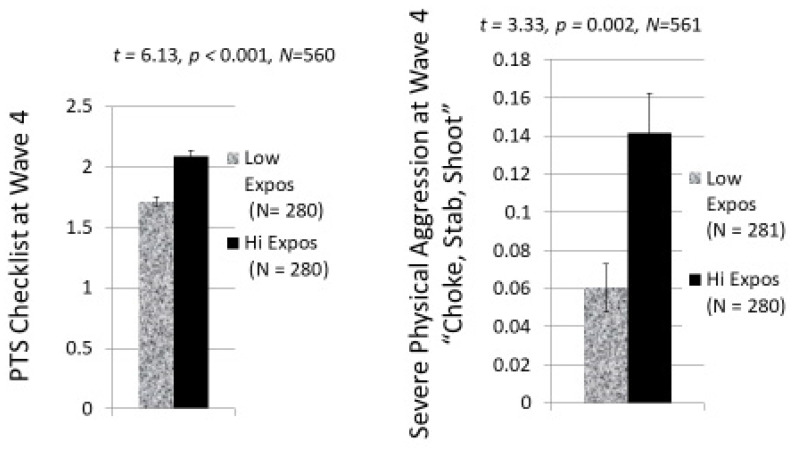
Mean levels of PTS Symptom Scores and Severe Physical Aggression Scores at age 14–20 for participants who were above (Hi Expos) or below (Low Expos) the median on cumulative exposure to ethnic-political violence 4 to 8-years earlier during Waves 1, 2, and 3.

Perhaps a better picture of the magnitude of these two significant relations is illustrated by the bar graphs in Figure 1, which show the mean PTS score and mean Severe Physical Aggression score for those who were above or below the median in exposure to ethnic–political violence in Waves 1–3. For example, one can see that those who are high in exposure to ethnic–political violence are on average about twice as likely to engage in choking, stabbing, or shooting 4 to 8 years later as those low on exposure.

**Figure 2:**
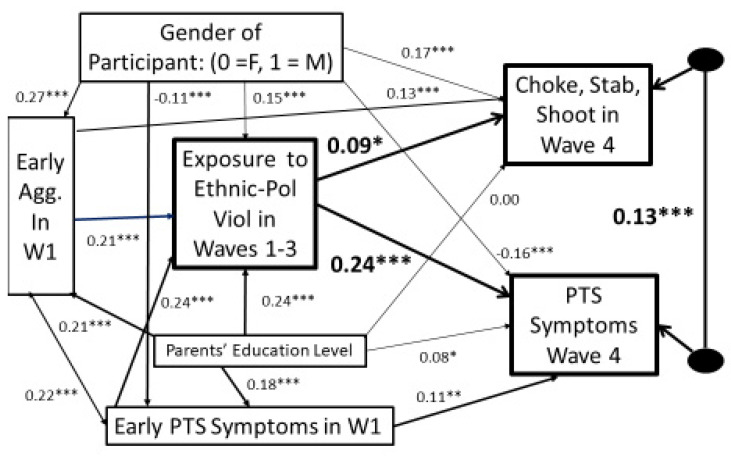
The longitudinal simultaneous prediction of severe physical aggressive behavior (logged) and PTS symptoms at age 14 to 20 from cumulative exposure to ethnic-political violence 4 to 8 years earlier during waves 1, 2, and 3 controlling for participant’s gender, early aggression, early PTS symptoms, and family’s education level (standardized coefficients). * *p* < 0.05. ** *p* < 0.01. *** *p* < 0.001. *N* = 1051; Chi-square = 0.32, *df* = 1, *p* = 0.57; *NFI* = 0.999; *RMSEA* = 0.

In Figure 2 we computed a structural model testing how the cumulative exposure to ethnic–political violence in Waves 1 to 3 predicts both severe physically aggressive behavior and the frequency of PTS symptoms 4 to 8 years later in Wave 4. We estimated the parameters of the model using Amos’s FIML estimation algorithm to correct for missing data, while controlling for the participants’ genders, early aggression, early PTS symptoms, and their parents’ education level. As Figure 2 shows, exposure to more ethnic political violence in Waves 1 to 3 independently predicts both more severe physically aggressive behavior and more PTS symptoms years later in Wave 4 independently of the effect of early aggressive behavior and early PTS symptoms.

Table 2 also reveals that in Wave 4 PTS Symptom scores are significantly positively correlated (*r* = 0.15, *p* < 0.001) with Severe Physical Aggression scores (choking, stabbing, or shooting). While this correlation is significant, it is not high in absolute value, suggesting that some participants score high on one and not the other. Furthermore, the significant residual relation between them that is shown in Figure 2 suggests that other factors contribute to what correlation there is. Given the significant longitudinal relations shown in Figure 2 between exposure to violence and both of these outcome variables, it follows that it is plausible that some people may have their severe physical aggressiveness affected more by exposure to violence, while other people have their PTS symptoms affected more by violence exposure. Our hypotheses, as described in the Introduction, are, first, that people with characteristically high anxious arousal to violence are in general less likely to behave aggressively and more likely to experience PTS symptoms, and, secondly, that people with high anxious arousal to violence are less likely to imitate what they see and have their aggression increased by exposure to violence.

**Figure 3:**
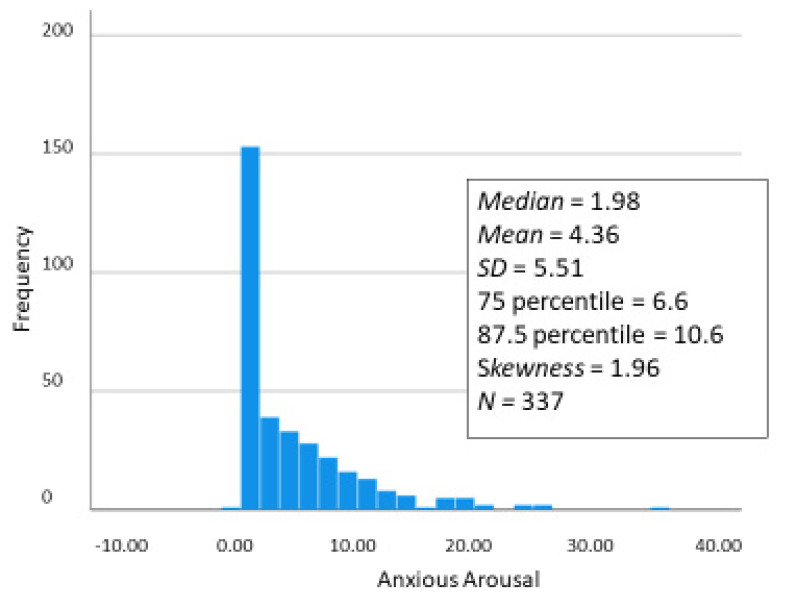
Frequency distribution of Participants’ “Characteristic Levels of Anxious Arousal to Violence” as assessed in Wave 4 when participants were age 14 to 20.

To test these hypotheses we assessed each participant’s characteristic Anxious Arousal to Violence with the procedure described in the Methods section. The obtained distribution of Anxious Arousal to Violence scores is shown in Figure 3.

The distribution is quite non-linear and skewed toward 0, with its median being 1.98 and its maximum being 35. Therefore, for comparing mean outcome scores for high-anxious-arousal people with low-anxious-arousal people, we denote those who scored below the 25th percentile as “low anxious arousal to violence” people and those who score at about the 75th percentile as “high anxious arousal to violence” people. According to our theorizing, the group of individuals higher in “anxious arousal to violence” on average should be *less* prone to behaving severely physically aggressively because thinking about violence makes them anxious, but they should be equally prone or more prone to experiencing PTS symptoms because of their characteristically higher anxious arousal to violence.

**Figure 4:**
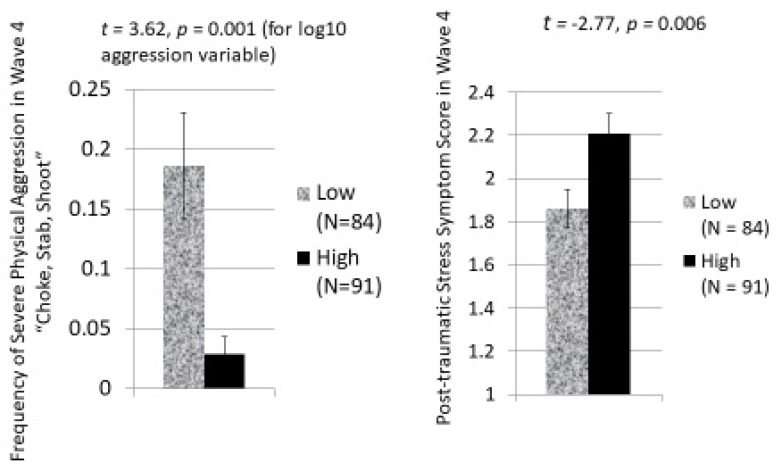
Mean levels of Severe Physical Aggression and PTS Symptom Scores at age 14 to 20 during Wave 4 for participants who are characteristically high (≥75 percentile) or low (≤25 percentile) on Anxious Arousal to Violence.

As the bar graphs in Figure 4 show, this is exactly what we found. Participants higher in anxious arousal have a mean score of Severe Physical Aggression in Wave 4 that is significantly lower than that of participants with lower anxious arousal, but participants with higher anxious arousal have a mean score on PTS symptoms that is significantly higher than other participants’ mean score.

**Figure 5:**
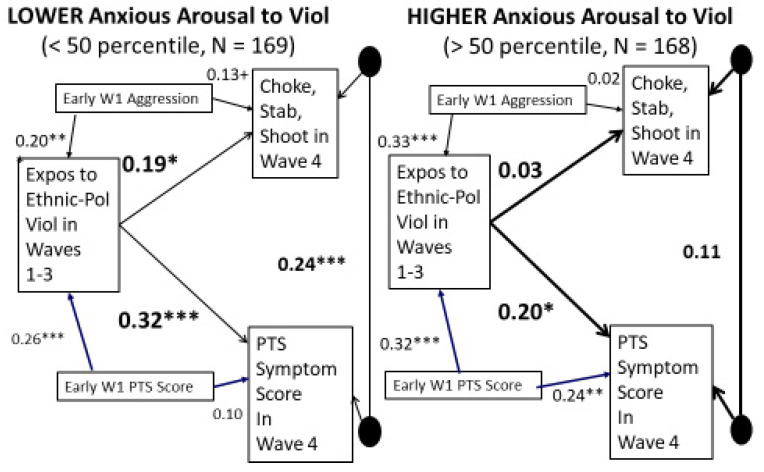
Moderation by Anxious Arousal of Effects of Exposure to Violence on Severe Physical Aggression (logged) controlling for prior aggression and on PTS Scores controlling for prior PTS scores while also controlling for the participant’s gender and family’s education level (not shown). + *p* < 0.10. * *p* < 0.05. ** *p* < 0.01. *** *p* < 0.001 Chi-sq = 7.81, *df* = 6, *p* = 0.252, *NFI* = 0.975, *RMSEA* = 0.03.

To test our hypothesis that this difference is at least partially due to participants’ characteristic level of anxious arousal to violence moderating the influence of exposure to ethnic–political violence on their subsequent severe physical aggression and PTS symptoms, we estimated the parameters of the two-group longitudinal moderation model shown in Figure 5, comparing participants with anxious arousal scores below the median with those with scores at about the median while controlling for their initial aggression and PTS symptoms, their gender, and their parents’ educational level. We used a median split instead of the 75th and 25th percentiles to test for moderation to utilize the whole sample and achieve a more powerful test.

The resulting best-fit parameters estimated with AMOS’s full information maximum likelihood procedure show that a person’s characteristic anxious arousal to violence does indeed moderate the effect of ethnic–political violence exposure on the outcomes. Exposure to ethnic–political violence significantly increases severe physical aggression for those who are low (below the median) in anxious arousal to violence (β = 0.19, *p* = 0.02), but not for those who are high in it (β = 0.03, n.s.). However, exposure to ethnic–political violence significantly increases PTS symptoms for everyone.

**Figure 6:**
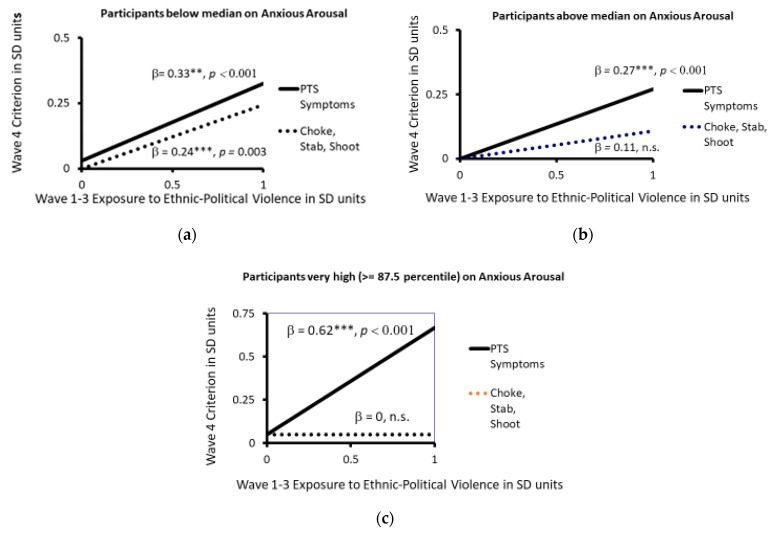
(**a**–**c**) The relation of participants’ PTS Symptom Scores and Severe Physical Aggression (‘Choke, Stab, Shoot’) in Wave 4 (ages 14 to 20) to their Cumulative Exposure to Ethnic-Political Violence 3 to 8 years earlier in Waves 1–3 for youth varying in their Characteristic Anxious Arousal to Violence: (**a**) (participants below the median on anxious arousal), (**b**) (participants above the median on anxious arousal, and (**c**) (participants at or above the 87.5th percentile on anxious arousal). ** *p* < 0.01, *** *p* < 0.001.

A more detailed elaboration of the moderation effect is shown in Figure 6a–c. Figure 6a shows the simple regression plots for both PTS symptoms and severe physical aggression on exposure to ethnic–political violence for those who are below the median in the level of their characteristic anxious arousal to violence. The standardized slopes of the regression lines are very similar. More exposure predicts more subsequent PTS symptoms and more subsequent severe physical aggression. Figure 6b shows the same plots for those who are above the median in their characteristic anxious arousal to violence. The standardized slope for exposure predicting PTS symptoms is very similar to the slope for those below the median in anxious arousal to violence, but the slope for predicting severe physical aggression from exposure is flatter and not significant, as we predicted for higher-anxious-arousal participants. Finally, Figure 6c shows the plots for the 42 participants who are very high in their characteristic anxious arousal to violence, i.e., in the top 12.5th percentile. For them, the slope for predicting severe physical aggression from exposure to ethnic–political violence is zero—that is, the amount of exposure has no relation to their subsequent severe aggression. However, for these very-high-anxious-arousal participants, the slope of the prediction line from exposure to PTS symptoms is 0.62 and highly significant. One can conclude from these analyses that a person’s characteristic anxious arousal to violence moderates the effects of exposure to ethnic–political violence on both subsequent PTS symptoms and subsequent severe physical aggression, particularly as the level of anxious arousal becomes extremely high or extremely low.

## 4. Discussion

While the results of this longitudinal, observational study cannot prove causal effects, the results show that more early exposure to ethnic–political violence for a youth predicts greater severe physically aggressive behavior (i.e., choking, stabbing, and shooting) by them 4 to 8 years later unless they display a high level of anxious arousal to violence. Additionally, more early exposure predicts more PTS symptoms 4 to 8 years later for most people regardless of their characteristic amount of anxious arousal to violence, but especially for those who have a very high characteristic level of anxious arousal to violence. These results were obtained even after controlling for participants’ gender, parental education, and participants’ earlier levels of aggression and post-traumatic stress symptoms.

Modern theories about the social–cognitive processes involved in observational learning [4,6,24,31] suggest that people encode the violent scripts they observe being used (particularly if they identify with the user) and employ the scripts later themselves, unless a script violates a normative belief or thinking about a script activates aversive emotions such as anxiety. As a person with high anxious arousal to violence is likely to experience negative affect when retrieving a violent script for solving a social problem, that person should be less likely to use such a script, and that is exactly what this study showed for people who were characteristically high in anxious arousal to violence. As the theory predicted, these individuals were significantly less likely to acquire and employ violent scripts from being exposed to ethnic–political violence.

Unfortunately, while this study suggests that high anxious arousal to violence may reduce the likelihood of an observer of ethnic–political violence learning and using a violent script, it also shows that they are no less likely and, if they have very high anxious arousal to violence, may be even more likely, to experience PTS symptoms after their exposure to violence. Their characteristically high anxious arousal to violence may well magnify the trauma of their exposure to ethnic–political violence, resulting in more PTS symptoms.

These findings present some problems for devising potential interventions for youths who are likely to be exposed to ethnic–political violence or war violence. If an intervention is aimed at decreasing youths’ anxious arousal from being exposed to violence and teaching psychological coping methods for dealing with it psychologically, it is possible that the intervention may decrease the occurrence of post-exposure PTS symptoms, but that may be at the expense of increasing the risk that a youth will learn the aggressive scripts that the youth has observed and therefore behave more aggressively afterwards. Our findings require replication, but if this is the case, the best solution may be to teach youths how to cope with their high anxious arousal to violence, and at the same time to prevent or reduce their aggression by training them to not encode aggressive scripts—but rather to promote non-aggressive social problem-solving scripts. Interestingly, this is the approach that has been adopted by some of the world’s most charismatic and successful non-violent peace promoters such as Martin Luther King, Jr., and Mahatma Gandhi.

### Limitations

As stated above, this kind of longitudinal observational study could not test conclusively whether exposure to ethnic–political violence actually causes subsequent aggressive behavior for those low in anxious arousal to violence, while it causes increased PTS symptoms for everyone but particularly for those high in anxious arousal. Testing the theory with a field experiment would provide more conclusive causal evidence but would raise some thorny ethical questions. As mentioned above, if one intervenes to reduce youths’ anxious arousal to observed violence, it is possible that this could increase their propensity to behave violently. On the other hand, if one intervenes to increase youths’ anxious arousal to observed violence, it is possible that this could decrease the violence-promoting effects of exposure, but with the downside of increasing the PTS symptoms the youths may experience after the exposure.

The other major limitation is that our sample size was too small to test whether the moderation model was equally valid for Jewish Israeli youths and Palestinian youths. Using the full information maximum likelihood procedure for estimating the parameters of the model while controlling for the gender of the youths, their earlier levels of aggression and PTS symptoms, and their parents’ education allowed us still to have reasonable power for our analyses, even with only a subset of the participants being evaluated for anxious arousal to violence. However, because ethnic group is confounded with exposure and aggressiveness (with much higher exposure and aggressiveness among Palestinian compared to Israeli youths [19]), we would have lost enough variance in our key measures to test the model validly had we computed separate models for each ethnic group.

## Figures and Tables

**Table 1:** Descriptive Statistics for the Study’s 4 Key Variables.

Variable	Mean	Median	Min	Max	SD	Skewness
Avg. Yearly Frequency of Exposure to Political Violence in W1–W3 (0 = never, to 3 = many times) *N* = 1051	0.81	0.82	0.00	1.90	0.36	0.13
Frequency of *Severe Physical Aggression* in W4 (0 = never, to 3 = 5 or more times) *n* = 561	0.10	0.00	0.00	2.67	0.29	4.05
Post-Traumatic Stress Symptom Score in W4 (1 = bothered by them not at all to 5 = extremely bothered) *n* = 560	1.90	1.76	1.00	4.76	0.76	0.92
Characteristic Anxious Arousal to Violence as measured in W4 (Theoretical range: 0 to 36) *n* = 337	4.36	1.98	0.00	35.18	5.50	1.96

**Table 2:** Correlations among Exposure to Ethnic–Political Conflict/Violence in W1–3, Severe Physical Aggression in W4, Post-Traumatic Stress Symptoms in W4, and Characteristic Anxious Arousal to Violence in W4.

Variable	Avg. Yearly Freq. of Exposure to Political Violence in W1–W3	Frequency of Severe Physical Aggression in W4	Post-Traumatic Stress Symptom Score in W4	Characteristic Anxious Arousal to Violence in W4
Avg. Yearly Frequency of Exposure to Political Violence in W1–W3	---			
Frequency of *Severe Physical Aggression* in W4	0.18 *** n = 561	---		
Post-Traumatic Stress Symptom Score in W4	0.25 *** n = 560	0.15 *** n = 560	---	
Characteristic Anxious Arousal to Violence as measured in W4	−0.11 * n = 337	−0.17 ** n = 337	0.13 * n = 337	---

Note. The *Severe Physical Aggression* variable in W4 was logged in this analysis because it was highly skewed. * *p* < 0.05. ** *p* < 0.01. *** *p* < 0.001.

## Data Availability

The data are not publicly available due to privacy and ethical restrictions. However, deidentified data used in this paper can be requested by appropriate researchers from the first author.
